# Effects of force level and task difficulty on force control performance in elderly people

**DOI:** 10.1007/s00221-020-05864-1

**Published:** 2020-07-13

**Authors:** Caren Strote, Christian Gölz, Julia Kristin Stroehlein, Franziska Katharina Haase, Dirk Koester, Claus Reinsberger, Solveig Vieluf

**Affiliations:** 1 Institute of Sports Medicine, Paderborn University, Warburger Str. 100, 33098 Paderborn, Germany; 2 Department of Psychology and Sports Science, Bielefeld University, Universitätsstraße 25, Bielefeld, 33615 Germany; 3 Faculty Business and Management, BSP Business School Berlin, Calandrellistr. 1-9, Berlin, 12247 Germany

**Keywords:** Force control, Elderly, Sensory motor task, Cognition, Entropy

## Abstract

As the proportion of people over 60 years of age rises continuously in westernized societies, it becomes increasingly important to better understand aging processes and how to maintain independence in old age. Fine motor tasks are essential in daily living and, therefore, necessary to maintain. This paper extends the existing literature on fine motor control by manipulating the difficulty of a force maintenance task to characterize performance optima for elderly. Thirty-seven elderly (*M* = 68.00, SD = 4.65) performed a force control task at dynamically varying force levels, i.e. randomly changing every 3 s between 10%, 20%, and 30% of the individual’s maximum voluntary contraction (MVC). This task was performed alone or with one or two additional tasks to increase task difficulty. The force control characteristics accuracy, variability, and complexity were analyzed. Lowest variability was observed at 20%. Accuracy and complexity increased with increasing force level. Overall, increased task difficulty had a negative impact on task performance. Results support the assumption, that attention control has a major impact on force control performance in elderly people. We assume different parameters to have their optimum at different force levels, which remain comparably stable when additional tasks are performed. The study contributes to a better understanding of how force control is affected in real-life situations when it is performed simultaneously to other cognitive and sensory active and passive tasks.

## Introduction

Advanced age is associated with changes in behavioral outcomes and the loss of multiple fundamental abilities that affect the individual’s quality of life. One important ability that decreases with age is fine motor control, which is essential to many activities of daily living and often has to be performed simultaneously with other tasks. Beside others, force control is crucial for performance in fine motor tasks. The aim of the current study was to characterize performance optima for elderly by manipulating the difficulty of a force maintenance task in terms of task difficulty.

Reaching and maintaining a certain force level depends on physiological and cognitive prerequisites. The musculoskeletal system and the cognitive control must be optimally coordinated, to achieve small highly accurate change in the produced force which causes a certain effect (Magill and Anderson 2017). Both are affected by aging processes resulting in performance loss. Regarding physiology and among other effects, a decreasing differentiation of the innervation of muscle fibers, repeating cycles of denervation and reinnervation of muscle fibers due to natural apoptosis of spinal motor neurons cause a higher innervation ratio per motor unit (MU). Fewer but larger MUs are the result (Hepple and Rice 2016; Hunter et al. 2016), which are characterized by an increased force per MU (Doherty and Brown 1997). Less smooth force adjustments, reduced maximal strength, decrease of contractile velocity, increases in fatigability and variability during and between motor tasks (Hunter et al. 2016) are the consequences. Additionally, decrements in underlying motor control processes (Voelcker-Rehage et al. 2006) as well as a reorganization of those (Temprado et al. 2017) have been associated with a reduction of force control performance. Moreover*,* degenerative processes concerning white matter and alterations of functional networks in the central nervous system lead to deficits in information processing and a reduced attention control ability (Johnson and Proctor 2004).

Hunter et al. (2016) suggested that elderly show an inability to appropriately regulate and coordinate the common synaptic inputs onto spinal motor neurons in attention-demanding situations, additional to difficulties to allocate and coordinate neuronal resources described by McDowd (1986). A consequence of these inabilities is a decline in fine motor control. After Hunter et al. (2016) increased variability in the force output of elderly people can be attributed to greater fluctuations in common oscillatory synaptic input compared to younger people. Temprado et al. (2015) showed that a decrease of attentional effort is detrimental for force control performance. Operating several tasks simultaneously (high task difficulty) requires attention control, and multiple task goals have to be maintained during task execution (Johnson and Proctor 2004). Elderly people experience more difficulty to redeploy attention among more than one task (Korteling 1991) and show a general slowing of information processing speed (Craik et al. 1994; Johnson and Proctor 2004). Salthouse and Somberg (1982) assumed this slowing of information processing speed in older adults to be independent of information type. Additionally, Voelcker-Rehage and Alberts (2007) summarized findings by Baltes and Lindenberger (1997), Kramer et al. (1995), Li and Lindenberger (2002) and Wingfield et al. (2005) on the central statement that elderly people need to pay a greater amount of attention to certain motor tasks to ensure a higher level of cognitive control and supervision, due to sensory loss, impaired sensorimotor performance and less efficient cognitive control processes, compared to younger people.

Force control describes the capability to adjust the force output along given constraints to achieve a certain goal (Temprado et al. 2017). Force control performance has been described in the past by three characteristics: Accuracy, variability and complexity. Accuracy is a measure of the error relative to the target force (Vaillancourt and Newell 2002). Variability describes to what extent the force output differs over time. In other words, it can be described as the standard deviation of the deviation from the target force and is, therefore, independent of the given target force. Complexity describes the structure of variability. The investigation of complexity of a force output is an opportunity to get insights into the organization of the underlying biological systems (Vaillancourt and Newell 2002), as it is an indicator for changes in time-structure of behavioral output fluctuations (Vieluf et al. 2015). As all three parameters are easy to measure, comparable and affected by described age-related changes, they were used for the investigation of force control in great number of studies in this field to characterize age-related differences (Papegaaij et al. 2017; Temprado et al. 2015; Vieluf et al. 2013; Voelcker-Rehage and Alberts 2007; Voelcker-Rehage et al. 2006).

With increasing age, accuracy and complexity are decreasing while variability is increasing (Temprado et al. 2017; Vaillancourt and Newell 2003; Vieluf et al. 2013). Moreover, experiments showed that different force levels relative to the individual’s maximum voluntary contraction (MVC) seem to be differently well controllable with a maximum performance around 20% of the individual’s MVC. In young adults, this maximum is characterized by higher accuracy as well as lower variability (Slifkin and Newell 1999). Additionally, age-related differences seem to be more pronounced, when additional tasks need to be operated simultaneously (Voelcker-Rehage et al. 2006).

Dual-task costs manifest in force control performance by a greater variability and lower accuracy (Voelcker-Rehage and Alberts 2007; Voelcker-Rehage et al. 2006). Temprado et al. (2017) revealed a reorganization of the interaction between preplanned and online control processes under different force control contexts. They assumed that behavioral slowing and overreliance on online control processes depend on the task itself. Additionally, Voelcker-Rehage et al. (2006) revealed a relationship between motor and cognitive performance under dual-task conditions. Elderly people showed a significant decrease in motor performance as well as in cognitive performance compared to the single-task condition, while the performance remained constant in the group of younger participants. Observing the phenomena that cognitive errors caused a greater variability in force control performance, Voelcker-Rehage et al. (2006) suggested that cognitive motor deficits are responsible for decrements under dual-task conditions. Consequently, the difficulties in attention control and slowing of information processing speed of cognitive control processes seem to play an important role in the understanding of force control performance in elderly people. Taken together, the contextualization of force control tasks plays an important role for the investigation of force control performance in fine motor tasks.

In the present study, we investigated if effects of force level remain stable across different task difficulties in elderly. We expect the 20% force level to be the one with the highest performance level, characterized by higher accuracy and complexity and lower variability. To answer this, participants had to perform a force control task with three different force levels (10%, 20% and 30% of their MVC). Task difficulty was increased by adding a secondary and tertiary task. Force control performance can be investigated using several characteristics of the force output. Here, accuracy, variability and complexity were used to characterize the force control performance. These parameters are also affected by above described physiological age-related changes in the way, that they cause a lower accuracy as well as a higher variability in the force output. A further decrease in force control performance with increasing task difficulty was expected. The investigation of the effect of both, force level and task difficulty, on force control performance can permit insights into the relation of those two factors. An interaction would indicate the varying importance of cognitive control processes at several force levels. A superiority of the 20% force level would support the assumption that force control at certain force levels needs less attention and is, therefore, less affected by higher task complexities.

## Methods

### Participants

As the focus of the study is on an age-specific problem, sample of this study consists exclusively of people with 60+ years of age. In total 38 participants were tested. One participant was excluded due to a significant higher ADAS score (ADAS score = 17) compared to the remaining sample (*M* = 7.59, SD = 2.71) of 37 participants (60–78 years; *M* = 68.00, SD = 4.65, see Table 1 for sample characteristics). They were recruited through newspaper announcements, announcements in the internet, as well as by personal contact in leisure activities for elderly people. The experimental protocol was approved by the ethics committee of the Ärztekammer Westfalen-Lippe and the Westfälische Wilhelms-Universität Münster as well as registered at the German Clinical Trials Register (DRKS00014921). Procedures were in accordance with the ethical standards laid down in the Declaration of Helsinki. All participants signed a written informed consent in advance. According to the Edinburgh Handedness Inventory (Oldfield 1971) all participants were right-handed. All participants declared to have normal or corrected to normal vision and hearing. None of the participants reported neurological, psychiatric or any other kind of disease that affects cognitive or motoric skills.

**Table 1:** Sample characteristics: Age and BMI for male and female (mean, SD)

	*N*	Age	BMI
Male	18	66.98 (4.50)	26.45 (2.74)
Female	19	69.05 (4.48)	25.87 (3.61)
Total	37	68.00 (4.65)	26.12 (3.19)

Presenting characteristics of the reduced sample of 37 participants, excluding one participant due to deviating ADAS scores

### Screenings

Before starting the actual measurement, participants underwent general and task-specific screenings to determine the performance corresponding to their age group. To be able to assess the cognitive and emotional level of the participants, they completed a battery of psychological tests: Becks Depression Inventory (BDI; Beck et al. 1961), Alzheimer's Disease Assessment Scale (ADAS, cognitive subscale, German adaptation; Rosen et al. 1984), Trail Making Test (TMT; Tischler and Petermann 2010; Rodewald et al. 2012), Corsi Block Tapping Test (Corsi 1972), Respond Inhibition Test (INHIB: Weisbrod et al. 2011), Regensburg Verbal Fluency Test (RWT: Aschenbrenner et al. 2000). The emotional level, in terms of depressive tendencies, were measured, due to the fact that depressive mood has a negative effect on short-term memory performance (Joormann and Gotlib 2008). A general discussion of the Trail Making Test can be found in Tischler and Petermann (2010) as well as one of the RWT in Aschenbrenner et al. (2000). The screening lasted maximal one and a half hours. Participants were asked to fill out the BDI at home. The screening started with the cognitive subscale of the ADAS followed by the RWT. Finally, participants completed TMT-L, CORSI and INHIB of the Wiener Testsystem (WTS, Version 6.82.000) provided by Schuhfried GmbH (Mölding). The last computer-based part of the cognitive screening was carried out independently by the participants.

The sample of the current study can be classified as active (according to the PASE questionnaire; Logan et al. 2013) and well educated (13.5 $\pm$ 4.2 years), which indicates a selection bias that may affect generalizability of the results. Task-specific screening comprised a motor, a tactile and a cognitive task. For the motor screening, the MVC was registered. Participants had to produce MVC in the precision grip position (using index finger and thumb) three trials, each lasting 5 s. The inter-trial interval lasted 60 s. The highest value was taken into account as the maximum value. Furthermore, the tactile threshold of each participant as well as the individual reaction time were determined. Tactile threshold was detected on the non-dominant hand on the fingertip as a two-point discrimination test and was achieved as soon as seven out of ten presented stimulations were correctly recognized. Reaction time was defined as the delay of the participants response to an acoustic stimulus, here 60 spoken letters. See Table 2 for screening results.

**Table 2:** Screening results: maximum voluntary contraction (MVC), tactile threshold, reaction time, Alzheimer’s Disease Assessment Scale (ADAS), Becks Depression Inventory (BDI), Physical Activity Scale for the Elderly (PASE), Regensburger Wortflüssigkeits-Test (RWT), Corsi Block Tapping Test (CORSI), Trail Making Test (TMT), Response Inhibition Test (INHIB)

	Min	Max	Mean	SD
MVC [*N*]	26	95	59.03	18.47
Tactile threshold [mm]	2	5	3.38	0.86
Reaction time [s]	0.68	1.00	0.84	0.08
ADAS	3	13	7.59	2.71
BDI score	0	16	3.06	3.77
PASE score	41	363	167.15	63.62
RWT semantic	12	50	33.38	7.15
RWT lexical	8	29	19.62	4.94
RWT semantic category	12	36	21.30	4.42
RWT lexical category	7	30	18.14	5.24
CORSI S1	0	6	4.47	1.00
CORSI S5	0	6	4.06	1.29
TMT A	16.00	41.50	24.72	4.88
TMT B	27.20	255.20	54.98	29.68
INHIB	0.26	0.63	0.37	0.06

### Setting and materials

#### Procedure

Each participant took part in an individual session. Participants had to perform a force control task under single (ST), dual- (DT) and multitask (MT) conditions (see below). Participants started with the single-task condition. Then the cognitive task (n-back task) and subsequently the tactile task (tactile oddball task) were added. After completing the multitask condition, the complexity of the overall task was reduced step by step by leaving out the secondary and tertiary task in reverse order. Each trial at each task difficulty level (ST, DT and MT) lasted 90 s.

#### Experimental setup

**Fig. 1:**
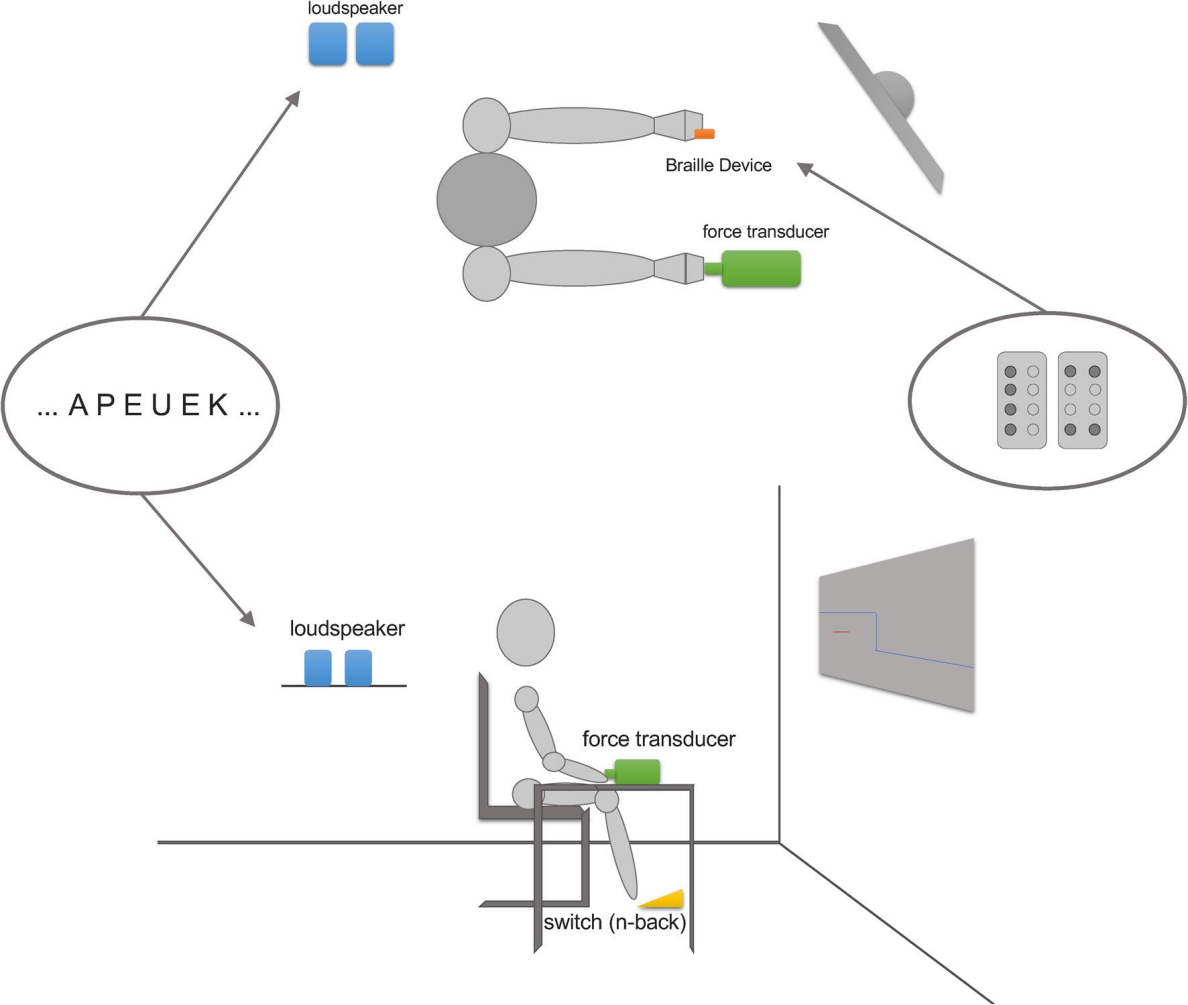
Illustration of the experimental setup. The upper part shows the top view and the lower part the same setup from a side view. Devices are highlighted in color and stimuli are depicted in the bubbles on the left and right

Participants were seated on a chair in front of a screen (AOC, 1920 × 1080 Pixels at 60 Hz, 23.8″) approx. 80 cm away. Their right wrist (dominant side) rested on a table, holding a force transducer between thumb and index finger (precision grip). Their left hand (non-dominant side) rested on their leg attached to a braille device (Braille-Modul B11, Metec AG, Stuttgart). The force transducer used for the force measurement worked with a platform load cell (1022-C3-20 kg, Soemer Messtechnik GmbH, Lennestadt), based on the strain gauge measuring principle. Analog to digital conversion as well as calibration was realized via a microcontroller (LDU68.1, Soemer) with amplitude resolution of 17 bit. Visual feedback as well as data input and output were implemented via RS-232 and Field Programmable Gate Array (FPGA, Xilinx XUPV5-LX110T Evaluation Platform) running custom build software. The experimental setup is illustrated in Fig. 1.

#### Force control task

The force control task itself required the force production at three force levels (10%, 20% and 30% of the individual’s MVC), which were displayed on a screen as a continuous blue target line. Participants were asked to follow that line by adjusting their force output manipulating the force transducer. Each force level was displayed for 3 s in a randomized order. Participants got visual feedback by a red bar displaying the induced force.

#### N-back task

A two-back task was realized as a listening task using recordings of random letter sequences. When the participants recognized the same letter as two letters back, they were asked to tap on a foot switch (StealthSwitch SS1R4 Pro USB, StealthSwitch), placed next to their right foot (Dobbs and Rule 1989; Li and Sikstrom 2002).

For the task, recordings of spoken letters of the German alphabet (except y, ä, ö, ü, ß) were sequentially presented by two loudspeakers placed approx. 50 cm behind the participant. Nine random sequences, each with 60 letters and a repetition rate of 20%, were generated before data acquisition. For each trial during data acquisition, a sequence was randomly chosen by a generator. Letter presentation lasted approximately 0.3 s per character and a break of 1.2 s was given between characters. Due to the 20% repetition rate, 12 responses were required per trial.

#### Tactile oddball task

The braille device (METEC AG Braille B11, metec Ingenieur-AG, Stuttgart) was fastened by one of the investigators to the left index finger (non-dominant hand). It was an eight-point braille module with a length of 52.6 mm and a width of 6.42 mm. The point spacing of the point distance is 2.45 mm and the point height is approximately 0.7 mm (for further information see https://www.metec-ag.de/produkte-braille-module.php?p=b11#). Two different patterns, either four pins on the left side (80% occurrence) or two pins on top and two at the bottom (20% occurrence) sticking out, were presented for a duration of 0.5 s (see Fig. 1). This ratio of eight to two is in line with the design of Reuter et al. (2012). Inter-stimulus interval varied randomly between 0.8 and 1.2 s (mean duration: 1 s), resulting in 60 stimulus presentations per trial on average. We chose a passive oddball task to not overwhelm the elderly participants. Thus, participants had to perceive but not respond to those patterns. To ensure that they do so, they were informed that they will have to describe the perceived figures afterwards.

### Data analysis

#### Preparation of force control data

The performance of an individual during a force control task can be described using the terms accuracy, variability and complexity. All behavioral parameters were calculated in relation to the individual’s MVC, so that the relative comparability of the values over the participants is given. In total, participants performed six blocks of force control tasks. One block lasted 90 s with 30 force steps of 3 s. The force levels were defined as 10%, 20% and 30% of MVC. Calculations were applied to intervals of 1.5 s in the middle of each force step to avoid ramp phase artifacts, after data were filtered offline using a lowpass Butterworth-filter up to 30 Hz. The first trial of each force level was considered as habituation period and therefore not included in the analysis. In the statistical analysis, the arithmetic-mean per parameter of all intervals of both blocks with the same task difficulty (first and second block) was calculated. Consequently, the parameter values used for statistical analysis are based on all intervals with the same target force level from both trials of 1.5 s each.

Accuracy was calculated using time within five percent range (TWR) as index. The value of the TWR parameter indicates the percentage of time within the range of the entire trial (Vieluf et al. 2013; Voelcker-Rehage and Alberts 2005, 2007). Variability was measured as the standard deviation of the deviation from the target force (SD). It is calculated in relation to the target force and represented in percentage (Temprado et al. 2017; Voelcker-Rehage and Alberts 2007). Therefore, the difference between the target force and the force output was calculated first. The standard deviation of this difference was calculated over each interval. Last, multi-scale entropy (MSE) was calculated as a measure of complexity. We set the tolerance range *r* to 0.2 and the number of data points *m* to 2, as recommended for physiological data (Costa et al. 2002, 2005; Gölz et al. 2018; Vieluf et al. 2018, 2015). It presents the fluctuations or structure of variability over time (for formula see Costa et al. 2005). Here, the mean over scales is reported. All scripts for data processing were written in MATLAB R2017b (MathWorks, Natick, MA, USA).

### Statistical analysis

For statistical analysis, IBM SPSS Statistics (Version 25, Armonk, NY: IBM Corp.) was used. At three force levels (10%, 20% and 30% of the individuals MVC) by three task difficulties (ST, DT, MT), repeated measures ANOVA was conducted to assess the effects of task difficulty and force level on force control performance. Including ADAS scores as a covariate in the analysis revealed no significant impact and were therefore not considered as covariate in the final model. Significant main effect and interactions were followed by Bonferroni corrected pairwise comparisons. An alpha level of 0.05 for all statistical tests was set a priori and 95% confidence intervals (95% CI) are presented. Effect sizes are given as partial Eta squares (*η*_p_^2^).

## Results

**Fig. 2:**
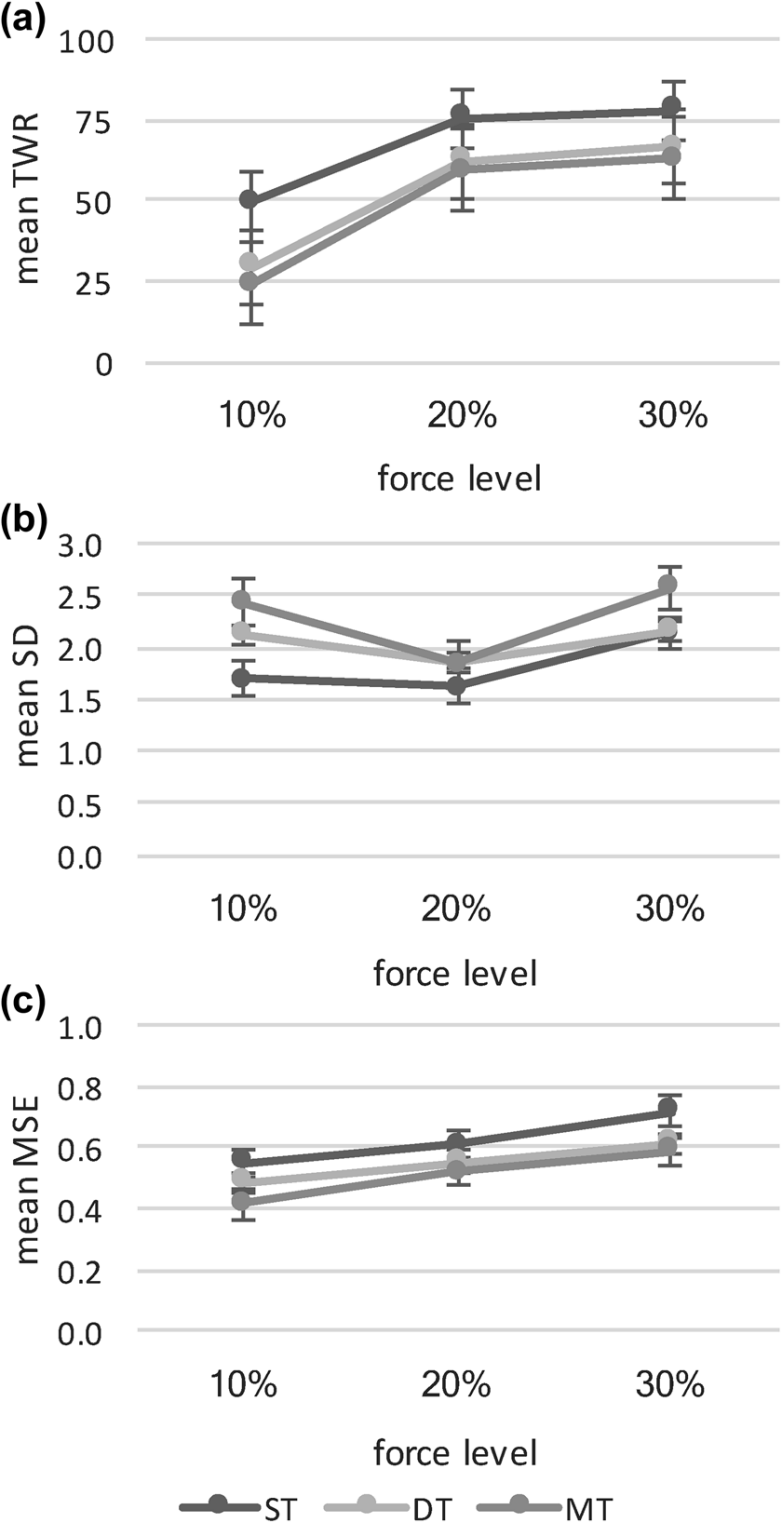
Means and standard errors are illustrated for **a** accuracy (mean TWR), **b** variability (mean SD), and **c** complexity (mean MSE) at each force level

Descriptive results are presented in Table 3 and Fig. 2. For TWR, main effects for both factors, force level (*F* (2, 72) = 94.115, *p* < 0.001, η_p_^2^ = 0.723) and task difficulty (*F* (2, 72) = 44.951, *p* < 0.001, η_p_^2^ = 0.555) were significant. The interaction of task difficulty and force level was not significant (*F* (4, 144) = 1.900, *p* = 0.125). Post hoc test with Bonferroni correction confirmed significant differences between ST and DT (*p* < 0.001; 14.89, 95% CI [9.58–20.20]) as well as ST and MT (*p* < 0.001; 18.70, 95% CI [13.85–23.55]), but not between DT and MT (*p* = 0.276). Further, differences between the force levels 10% and 20% (*p* < 0.001; 31.44, 95% CI [25.38–37.51]) as well as 10% and 30% (*p* < 0.001; 34.84, 95% CI [25.99–43.68]) were significant, but not between 20% and 30% (*p* = 0.446).

**Table 3:** TWR [% of time], SD [% of force], MSE: descriptive statistics (mean, SD)

Task difficulty	Force level	TWR		SD		MSE	
		Mean	SD	Mean	SD	Mean	SD
ST	10%	49.30	19.87	1.70	2.30	0.55	0.21
	20%	75.50	14.26	1.61	1.88	0.61	0.21
	30%	77.66	19.84	2.13	2.84	0.72	0.35
DT	10%	29.22	16.96	2.12	1.66	0.48	0.17
	20%	62.11	22.24	1.84	1.66	0.55	0.18
	30%	66.46	24.06	2.15	1.75	0.61	0.30
MT	10%	24.08	14.61	2.42	1.71	0.42	0.17
	20%	59.30	20.39	1.85	1.42	0.52	0.19
	30%	62.98	25.35	2.57	2.26	0.59	0.27

For SD, calculated as the standard deviation of the deviations, main effect of force level was significant (*F* (2, 72) = 5.339, *p* = 0.008, *η*_p_^2^ = 0.129) but not for task difficulty (*F* (2, 72) = 1.023, *p* = 0.343). Interaction was also not significant (*F* (4, 144) = 0.677, *p* = 0.596). Post hoc test with Bonferroni correction confirmed a significant difference between the 20% and 30% force level (10% vs. 20%: *p* = 0.172; 10% vs. 30%: *p* = 0.742; 20% vs. 30%: *p* = 0.003, 0.52, 95% CI [0.16–0.88]).

For MSE, main effects were significant for both factors, force level (*F* (2, 72) = 17.436, *p* < 0.001, *η*_p_^2^ = 0.326) and task difficulty (*F* (2, 72) = 6.054, *p* = 0.004, *η*_p_^2^ = 0.144). The interaction was not significant (*F* (4, 144) = 0.412, *p* = 0.762). Post hoc test with Bonferroni correction confirmed significant differences between ST and MT (*p* = 0.010; 0.12, 95% CI [0.02–0.21]) but not between ST and DT (*p* = 0.085) or DT and MT (*p* = 0.623). Furthermore, differences between all force levels were significant (10% vs. 20%: *p* = 0.001, 0.08, 95% CI [0.03–0.13]; 10% vs. 30%: *p* < 0.001, 0.16, 95% CI [0.08–0.24]; 20% vs. 30%: *p* 0.012, 0.08, 95% CI [0.02–0.15]).

## Discussion

Based on the assumption that in daily living, fine motor tasks are most often performed with other tasks simultaneously, the aim of this study was to investigate how force control characteristics change with increasing task difficulty at various force levels. Task difficulty was manipulated by the combination of the force control task with a cognitive or a sensory and a cognitive task. Analysis of accuracy, variability and complexity of the force output revealed that the higher the task difficulty was, the lower was the accuracy and complexity of the force output. Accuracy and complexity increased with increasing force level, while lowest variability was observed at the force level of 20% of the individuals MVC. Which confirms, in line with previous studies (Slifkin and Newell 1999), an optimum for variability at 20% of the individual’s MVC. No interaction effect between force level and task difficulty was found for variability. Considered in combination, this indicates that the force control performance characteristics react differently to requirements of force control tasks.

### Age-specific physiological properties affect force control performance

Besides cognitive declines of elderly, physiological changes are affecting the force control performance (Hunter et al. 2016). For example, an increased fatigability (Hunter et al. 2016) needs to be taken into account by planning force maintenance tasks. As it is necessary to do several trials with several repetitions of each force level, force level should be selected so that effects of fatigue should be minor. That is why we decided for low force levels, beside a randomized sequence of force levels. This prevented both fatigue and learning effects. Moreover, absolute force levels involve the risk that they are difficult to reach for different people. To ensure that each force level represents the same degree of difficulty for each participant, relative force levels were used in the present study.

Furthermore, decreasing differentiation of the innervation of muscle fibers and increasing MU sizes (Hunter et al. 2016) affects the force output characteristics. These changes have a direct impact on force modulation itself and, therefore, on measured force control performance. Increasing MU sizes cause greater changes in the force output leading to high variability in the force output. Therefore, variability of the force output should only be compared within a group of people at the same age. As the focus of the study is an age-specific description of force control characteristics and not the aging process, the sample of this study consisted of people over 60. The influence of physiological factors on force control performance compared to the one of cognitive has not been determined, yet. As described above, increased variability in force output can be attributed to cognitive restrictions, but it is also conceivable that the required force levels are more difficult to modulate for elderly. The increased MU size means that switching on and off individual MUs leads to greater changes in force output than in younger people due to greater forces per MU (Doherty and Brown 1997; Hepple and Rice 2016; Hunter et al. 2016). Therefore, elderly people have more difficulties to fine tune their strength. This finetuning difficulties also affects the accuracy of the produced force.

Moreover, findings by Vieluf et al. (2015) suggest that MU recruitment and complexity are related. The number of recruited MUs increases up to approx. 40–50% of the individual’s MVC. Consequently, force modulation depends on firing rates. This force level is close to their measured maximum of complexity and slightly above the force level range used in the current study. This could explain the increasing complexity with increasing force level and would support the assumption that different parameters have different optima in relation to the force level. Vieluf et al. (2015) showed that different force control parameters have different optima. In their study complexity had an optimum at 60% of the individual’s MVC. They also showed that parameters are affected differently by the force level. The complexity of the force output is increasing with increasing force level instead of an inverted U-shape as it is described for variability. To avoid fatigue, we did not investigate force levels higher than 30% in the present study.

Based on the observation of different optima for different parameters over force levels in past and our study, one could assume that this characteristic is important for the system's adaptability and flexibility. As the motor control performance includes different dimensions with different optima, this could enable the system to maintain a functional level over a wider range of force levels. That seems to be reasonable in the context of daily living, were people have to deal with not self-selected forces while solving fine motor tasks. Beyond pure physiological effects, a cognitive involvement in the specifics of force control must be assumed based on our data, too.

### The impact of cognitive control on force control in elderly

In contrast to other studies (Temprado et al. 2015; Zijdewind et al. 2006), we focused on changes in force control characteristics and did not measure other attentional markers. However, performance changes in force control tasks relate to attentional control processes and therefore allow to infer about those. For example, the decrease in performance with increasing task difficulty indicates an increase in attentional load.

Results showed that with increasing task difficulty force control performance decreased. Accuracy is affected continuously by increasing task difficulty, while complexity shows a significant difference between the single-task condition and multitask condition only. Decreasing accuracy with increasing task difficulty can relate to both, attention control deficits and information processing slowing. As the tactile task itself was not designed with a high cognitive load, integration of sensory feedback due to general information processing slowing might play a more important role concerning accuracy decrements than attentional control. Moreover, past studies (Papegaaij et al. 2017; Voelcker-Rehage and Alberts 2007) have shown, that younger people are less affected by an additional task than older ones. Together with the findings of Voelcker-Rehage and Alberts (2007) and Voelcker-Rehage et al. (2006), the assumption of force level and task difficulty as two factors is an adequate hypothesis that is supported by the present results. They demonstrated on the one hand, an interference of cognitive and motor performance under dual-task condition and, on the other hand, a positive effect of motor practice on force control performance. In Voelcker-Rehage and Alberts (2007), it is also described that motor practice does not prevent a decline in motor performance but cause an improvement in the cognitive task. An interrelation with attention and attention control-dependent task difficulty could combine all described findings.

### Limitations and outlook

Within this study, not all influencing factors could be captured or controlled. For example, the individual training level for each task type or motivation has not been controlled. However, selected analysis procedure was based on often described parameters. The study design in the current study plays an important role for the described transfer of results into real-life situations. The block wise design has two important advantages. It allows the participant to get used to task and situation step by step and draws the attentional focus to the force control task as primary task. The pyramidal structure of the blocks enables the fatigue effects to be reduced, as task difficulty decreases at the end. The randomization of the force level within the blocks ensures a higher comparability of the force levels by eliminating effect of the prior force level. That means, that the difference between successive force levels was averaged. Moreover, this design requires a dynamic adaptation of the force output over time, which provides a closer relationship to real situations. This allows an argumentation that supposes effects of automatization processes concerning force control for certain force levels in certain situations.

Moreover, in the past, some studies have been investigating different force levels or force levels that differed more from each other than the ones in this paper (Temprado et al. 2015) or used different designs of target line characteristics. Some papers ask for one force level per trial (Temprado et al. 2017; Vieluf et al. 2015) instead of a sequence of several force level per trial, as it was done in the current study. However, the design of the current study with three close and low force levels allows more detailed suggestions about the development of accuracy, variability and complexity over force levels at this low force level range, which is of interest for fine motor tasks. Additionally, analyzing sequences of force levels creates a closer relation to real-life situations, which corresponds to what our design aimed for. Furthermore, the use of a multitask condition, to investigate the impact of the situational complexity, is new. Results showed that performance decreased further due to the second additional task even though it has no significant cognitive load, as it was a passive sensory task.

In future studies, it could be of great interest to investigate a wider range of force levels on one hand, and the possible differences in the influence of tasks with high and low cognitive load on the other hand. These design elements could help to describe the exact form of the influence of task difficulty and force level on force control performance. The existence of one or several force level optima could be confirmed as well as a possible dependence of complexity effects on task type of additional tasks. Additionally, ideal additional tasks should be comparable in the level of difficulty to permit a steady increase of task difficulty. In the current study, task difficulty was operationalized as complexity of the situation, while difficulty, here, refers to the difficulty of the individual task. As number of output options is limited as well as the types of tasks per output option, such ideal additional tasks are more hypothetical then practical. Therefore, task difficulties should be assessed through several criteria that still need to be determined to achieve greater comparability.

If future studies confirm our assumptions, findings could be the basis for new training concepts. Past studies (Keogh et al. 2007, 2010) have already shown, that strength training improves force control performance. Now, knowing optima of force levels for certain task types, it might be possible train the strength to a level that the optima falls into the range of tasks of daily living, that are well controllable for elderly also in more complex situations.

## Conclusion

Taken together, our results confirm an optimal task performance level at 20% of MVC for variability of the force output in elderly. At this force level, the variability of the force control remained stable, when other tasks are performed simultaneously. Additionally, together with results from previous studies, our results support the assumption that different optima could exist for different parameters. This assumption could indicate a dynamic interaction of the underlying control process for the maintenance of an overall functional force control performance over a wider range of relevant force levels. However, as the single-task condition comes with the best force control performance, the attentional focus seems to be crucial for force control performance in the elderly. Referring to our argumentation and results, it speaks for a crucial role of cognitive resources for force control performance. However, parameter-specific optima seem to be robust for all three difficulty levels, single-task, dual-task and multitask. The chosen study design enabled a comparison of force control performance at three low force levels over three different task complexities, including multitasking.
